# Genome-Wide Association Study Suggests the Variant rs7551288*A within the *DHCR24* Gene Is Associated with Poor Overall Survival in Melanoma Patients

**DOI:** 10.3390/cancers14102410

**Published:** 2022-05-13

**Authors:** Annette Pflugfelder, Xuan Ling Hilary Yong, Kasturee Jagirdar, Thomas K. Eigentler, H. Peter Soyer, Richard A. Sturm, Lukas Flatz, David L. Duffy

**Affiliations:** 1 The University of Queensland Diamantina Institute, The University of Queensland, Dermatology Research Centre, Brisbane, QLD 4102, Australia; x.yong@uq.edu.au (X.L.H.Y.); kjagird1@jhu.edu (K.J.); p.soyer@uq.edu.au (H.P.S.); r.sturm@uq.edu.au (R.A.S.); david.duffy@qimrberghofer.edu.au (D.L.D.); 2 Center of Dermatooncology, Department of Dermatology, University of Tübingen, 72076 Tübingen, Germany; lukas.flatz@med.uni-tuebingen.de; 3 Clem Jones Centre for Ageing Dementia Research, The University of Queensland, Brisbane, QLD 4072, Australia; 4 Queensland Brain Institute, The University of Queensland, Brisbane, QLD 4072, Australia; 5 Biochemistry and Molecular Biology Department, Johns Hopkins Bloomberg School of Public Health, Baltimore, MD 21205, USA; 6 Department of Dermatology, Venereology and Allergology, Charité—Universitätsmedizin Berlin, Corporate Member of Freie Universität Berlin and Humboldt-Universität zu Berlin, 10177 Berlin, Germany; thomas.eigentler@charite.de; 7 Department of Dermatology, Princess Alexandra Hospital, Brisbane, QLD 4102, Australia; 8 Genetic Epidemiology, QIMR Berghofer Institute of Medical Research, Herston, QLD 4006, Australia

**Keywords:** melanoma, survival, GWAS, genome-wide association study, *DHCR24*, seladin-1, cholesterol

## Abstract

**Simple Summary:**

The aim of this work was to investigate prognostic genetic factors in melanoma patients. Phenotypic and disease data as well as biomaterial were collected after informed consent from patients followed up in a Skin Cancer Center of a University clinic. Genome-wide analysis (GWAS) was performed with survival data of 556 melanoma patients and genetic data including more than 300,000 common polymorphisms. The SNP rs7551288 reached suggestive genome-wide significance (*p* = 2 × 10^−6^). This intronic variant of the *DHCR24* gene is involved in the cholesterol synthesis pathway. Further analyses and a literature review suggest an important role of this locus for the clinical course of disease in melanoma patients.

**Abstract:**

Melanoma incidence rates are high among individuals with fair skin and multiple naevi. Established prognostic factors are tumour specific, and less is known about prognostic host factors. A total of 556 stage I to stage IV melanoma patients from Germany with phenotypic and disease-specific data were analysed; 64 of these patients died of melanoma after a median follow-up time of 8 years. Germline DNA was assessed by the HumanCoreExome BeadChip and data of 356,384 common polymorphisms distributed over all 23 chromosomes were used for a genome-wide analysis. A suggestive genome-wide significant association of the intronic allele rs7551288*A with diminished melanoma-specific survival was detected (*p* = 2 × 10^−6^). The frequency of rs7551288*A was 0.43 and was not associated with melanoma risk, hair and eye colour, tanning and total naevus count. Cox regression multivariate analyses revealed a 5.31-fold increased risk of melanoma-specific death for patients with the rs7551288 A/A genotype, independent of tumour thickness, ulceration and stage of disease at diagnoses. The variant rs7551288 belongs to the *DHCR24* gene, which encodes Seladin-1, an enzyme involved in the biosynthesis of cholesterol. Further investigations are needed to confirm this genetic variant as a novel prognostic biomarker and to explore whether specific treatment strategies for melanoma patients might be derived from it.

## 1. Introduction

Melanoma has high incidence rates among fair-skinned populations and is still a deadly cancer once metastasised. The predominant localisation of melanoma is the outer skin reflecting the exceptional vulnerability to the main carcinogen, UV radiation, but also the ideal suitability for early detection [1]. Melanomas most often arise from single melanocytic cells but also evolve from existing naevi, which are benign conglomerations of melanocytes in the skin [2]. As a tumour also affecting young adults, melanoma is among those cancers with the most substantial impact on years of life lost [3]. Incidence rates are still rising globally [4]. So far, environmental, phenotypic and genetic risk factors have been described for melanoma development. UV exposure is the only known environmental risk factor, and both UV exposure and genetic variants influence the number of melanocytic naevi, which is the most important independent melanoma risk factor [5]. Further established independent risk factors are the number of large or clinically atypical naevi, hair colour, skin type, a positive family history, freckling and a history of sunburns, as described in a risk algorithm tool based on a pooled dataset from 16 case–control studies [6]. Several genome-wide association studies (GWAS) on melanoma risk have been conducted in recent years and have identified an increasing number of risk loci [7,8,9,10,11,12,13,14,15]. Some of these melanoma risk loci are independent from host pigmentation traits and have been described in other cancer entities as well, such as variants within *TERT,* which are associated with longer telomere length [16], or variants of the gene *CDKN2A,* which are involved in tumour suppression [17]. Most of the retrieved melanoma risk loci, however, e.g., variants within the genes *MC1R*, *SLC45A2*, *ASIP*, *MTAP*, *IRF4* and *TYR*, are associated with pigmentary traits or total naevus count (TNC) [18].

The reported incidence and mortality rates of melanoma vary greatly among different global regions and ethnicities. While in some world regions, such as Middle Africa, nearly all melanoma patients die of their disease, in other world regions, the vast majority of patients do not develop metastases after the excision of the primary tumour. This is mostly seen in those areas with the highest incidence rates [19]. Studies on melanoma risk have been mainly performed on cohorts of populations with high incidence rates including a substantial rate of low-risk melanomas. Melanoma deaths are caused by metastases, which are rarely evident at primary diagnoses and develop typically with a latency of 1–3 or more years [20]. Prognostic factors for disease progression and melanoma survival have been established so far on tumour-specific characteristics, such as tumour thickness and ulceration. These factors contribute to the latest AJCC staging classification [21]. A focus on melanoma survival instead of melanoma development for genetic risk analyses might be suitable to select and classify patients who are at risk of developing metastasis. This study was carried out to find host-specific genetic prognostic markers in order to identify patients at risk of melanoma death. This might allow improved patient surveillance and management in the future.

## 2. Materials and Methods

### 2.1. Patient Recruitment and Measures

To conduct the present survival analyses, a cohort of 556 melanoma patients from Tübingen, Germany was drawn from a case–control study, “hereditary effects in malignant melanoma”. The control group of patients without melanoma was not included for the present analyses. The study was approved by the ethics committee of Eberhard Karls University in December 2007 (ethics number 376/2007B01). The first participant was included in 2007 and the last one in 2011. All study participants gave written informed consent prior to study entry.

Study participants were recruited and investigated by physicians during their regular appointments at the melanoma outpatient clinic. The patients completed a questionnaire covering information on pigmentation traits, response to sun exposure, personal sun behaviour as well as personal and family history of melanoma or other cancers. The total naevus count (TNC) was recorded by the physicians after physical examination and by the patients as a self-recorded estimate. A blood sample was taken from each patient for DNA analyses.

All melanoma patients were part of the Central German Melanoma Registry after written informed consent. The Central German Melanoma Registry recorded melanoma-specific data, such as the excision date of the primary melanoma, localisation, histology and initial stage of disease as well as date and localisation of subsequent metastases, subsequent clinical stages, date and cause of death. Data of the patients were prospectively obtained by the responsible physicians and entered by research assistants of the Central German Melanoma Registry.

### 2.2. Genotyping

Genomic DNA was derived from 2 mL EDTA whole blood samples, which were stored at −20 °C. The DNA extractions were performed with the column-based QIAamp DNA Blood Midi Kit (Qiagen, Inc., Valencia, CA, USA) according to the manufacturer’s instructions. Purified DNA was eluted from the affinity column with Buffer AE using volumes between 300 and 500 uL. The total yield per sample ranged between 2.9 and 140 μg of DNA. To obtain the required DNA concentrations of the genotype assays, a total of 372 samples were processed with a centrifugal evaporator (Savant Instrument, Inc., Farmingdale, NY, USA, model no. SVC 100 H). The DNA quality and concentration were measured using a spectrophotometer (NanoDrop™, Thermo Scientific, Waltham, MA, USA). Genotyping was performed using the Illumina HumanCoreExome-24 Chip array by the UQ Centre for Clinical Genomics (UQCCG) at the Translational Research Institute (TRI). A minimum of 2.5 μg of DNA was provided. The samples were submitted at a concentration range of 100 to 300 ng/uL, aliquoted in 96-well semi-skirted PCR Plates (Axygen Scientific, Union City, CA, USA) and sealed with Clear Self-Adhesive Topseal (PerkinElmer, Akron, OH, USA). Genotyping results were provided in a binary format (“bed”, “bim”, “fam” files). Study-participant genotyping success rates were 98.3% on average (minimum 97.2%). We removed monomorphic SNPs, those with a Hardy–Weinberg *p*-value < 1 × 10^−5^, and retained rarer SNPs, pruning only those where one alternative allele was observed in the entire sample, as empirically these did lead to spuriously significant survival association results.

### 2.3. Statistics

Genotypic data were extracted with the open-source C/C++ tool Plink [22]. Questionnaire data, registry data and genotypic data were entered and analysed using the statistical package SPSS (IBM statistics version 21) and Sib-pair (© by David Duffy, Version 1.0 beta). The genome-wide association study was performed with Sib-pair using the log-rank test with gene-dropping based empirical *p*-value estimation. The analysed time was defined between the date of first diagnoses and date of the last follow up. Melanoma deaths were recorded as events. Censored cases were patients who died of another reason and patients who were alive at the time of the last follow up. Survival probabilities were calculated using the Kaplan–Meier method. Survival plots were generated with Stata (StataCorp, College Station, TX, USA). An association test of the phenotype variables was performed using a two-sided Pearson Chi-Square test. Cox proportional hazard models were used to estimate hazard ratios (HRs) and their 95% confidence intervals (CIs). Patients with missing data were excluded. Multivariate analysis was performed using a forward stepwise method, *p*-values of< 0.05 were considered significant.

## 3. Results

**Figure 1:**
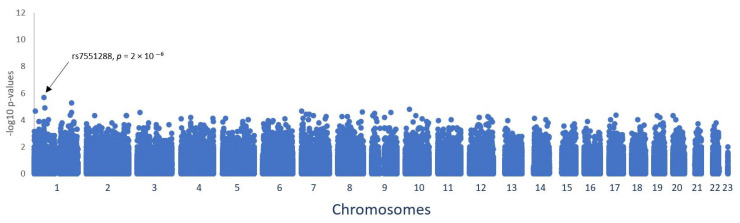
Manhattan plot illustrating the genome-wide association results of survival analyses in 556 melanoma patients from Germany. Each dot represents the −log10 *p*-value of the log rank test of the genotyped polymorphisms in association to the survival time of the patients. The dots are displayed on the *x*-axis according to the position of the polymorphisms on the 23 chromosomes. The SNP rs7551288 reached suggestive genome-wide significance with a *p*-value of 2 × 10^−6^.

A cohort of 556 melanoma patients from Germany and genetic data of 356,384 common polymorphisms of each patient were available for analyses. A genome-wide association study was carried out to investigate the impact of each single polymorphism on overall survival. The statistical significance levels were visualised by a Manhattan plot (Figure 1). Each dot corresponds to the *p*-level of a single polymorphism (*y*-axis) and to its chromosomal location (*x*-axis). A post-analysis investigation of the top hits was performed and significant results driven by single events were deselected. The Bonferroni level of significance (*p* < 5 × 10^−8^) was reached by none of the remaining polymorphisms. The threshold for a suggestive association which requires further validation was set at *p* < 5 × 10^−6^. The polymorphism rs7551288, located at chromosome 1, reached this suggestive genome-wide significance level with *p* = 2 × 10^−6^.

**Figure 2:**
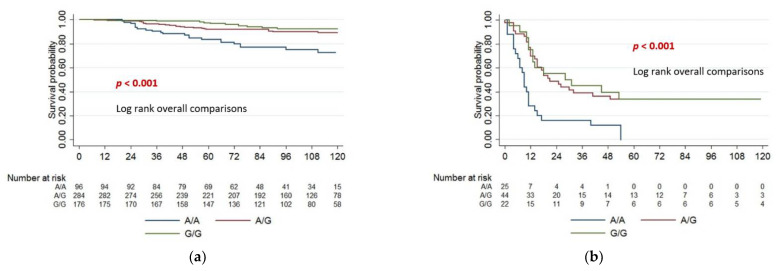
Kaplan–Meier curves stratified for the three different genotypes at rs7551288, patients homozygous for the rs7551288*A allele (A/A), heterozygous (A/G) and homozygous for the rs7551288*G allele (G/G). Survival probabilities were calculated from (**a**) date of primary diagnoses and (**b**) date of stage IV diagnoses until last date of follow up, time is given in months.

The polymorphism rs7551288 is a single-nucleotide polymorphism (SNP) with a single base change from guanine to adenine (G/A) in an intronic region of the *DHCR24* gene. In the European population, the rs7551288*G allele has a higher frequency than the rs7551288*A allele. There are three different genotypes, the homozygous genotypes GG and AA and the heterozygous genotype GA with a rs7551288*G allele and a rs7551288*A allele. The minor allele frequency (MAF) of rs7551288*A was 0.43 in the assessed cohort from Germany. The rs7551288*A frequency was between 0.37 and 0.43 in European populations, between 0.75 and 0.87 in Asian populations, and between 0.79 and 0.88 in African populations as reported in the dbSNP database. The Kaplan–Meier survival curves shown in Figure 2 revealed significant differences in survival probabilities between patients according to their rs7551288 genotype (*p* < 0.001). Patients with two rs7551288*G alleles (G/G) had the best survival probabilities (green line), patients with two rs7551288*A alleles (A/A) had the worst outcome (blue line) and heterozygous patients (G/A) were in between (red line). Survival probabilities were calculated for the whole cohort of 556 melanoma patients starting at the date of primary diagnoses (Figure 2a), the time was given in months (*y*-axis). A total of 64 melanoma-specific deaths were recorded during follow up. Of the 556 patients, 92 patients developed distant metastases and entered stage IV and 1 patient died in the month of stage IV diagnosis. Survival probabilities were calculated for the subgroup of 91 stage IV patients (Figure 2b). The Kaplan–Meier curve demonstrates again a significant impaired survival for stage IV patients with two rs7551288*A alleles (A/A, blue line), log rank overall comparison, *p* < 0.001.

**Figure 3:**
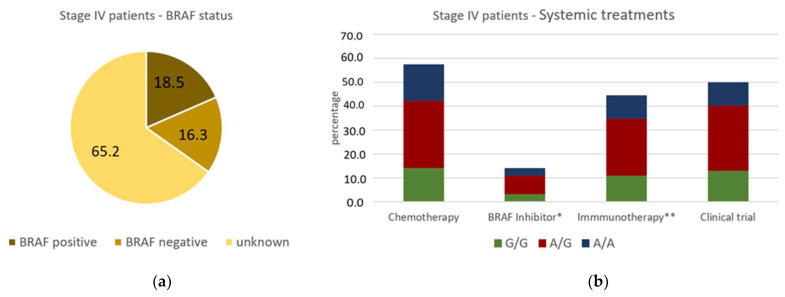
Details of the 92 patients who entered stage IV disease: (**a**) percentage of patients with positive, negative or unknown BRAF status; (**b**) percentage of patients receiving chemotherapy, BRAF inhibitor treatment*, immunotherapy** and systemic treatment within a clinical trial in stage IV. The share of the different genotypes is presented in green G/G, red A/G and blue A/A. * the unselective inhibitor Sorafenib was not considered as BRAF inhibitor treatment. ** this included RNA vaccination, L19-IL2 treatment, anti CTLA4 and PD1 antibodies.

During the recruitment period, new treatment options emerged for metastatic disease and melanoma survival increased with therapies such as immune-checkpoint inhibitors or BRAF inhibitors for patients with BRAF-positive melanomas. We therefore assessed survival times according to the systemic treatments the patients received. The BRAF status was known for 32 patients (34.8%) in our cohort of stage IV patients; 18.5% were BRAF positive and 16.3% were BRAF negative (Figure 3a). Half of the patients received systemic treatment within a clinical trial, 57.6% received chemotherapy, 14.1% received BRAF inhibitor treatment and 44.6% received immunotherapy, which included anti CTLA4 and PD1 antibodies as well as other immunotherapies within clinical trials (Figure 3a). Most patients received more than one systemic treatment. The three genotypes G/G, A/G and A/A were evenly distributed among the different treatment groups. The median overall survival in stage IV was 15 months for the whole cohort, 31 months for patients with G/G genotype, 21 months for patients with A/G genotype and 9 months for patients with A/A genotype. Patients who received BRAF inhibitors had a median survival of 31 months compared to 14 months for patients who did not receive BRAF inhibitors. Patients with the A/A genotype who received BRAF inhibitors had a median survival of 14 months compared to 9 months for those without BRAF inhibitor treatment. However, due to small numbers, these results must be treated with caution. Patients who received immunotherapy had a median survival of 18 months compared to 11 months for patients without immunotherapy. Patients with genotype A/A and immunotherapy had a median survival of 15 months compared to 7 months for those without immunotherapy. Patients with the genotype A/A who received chemotherapy had a median survival of 9 months; those who received no chemotherapy had a median survival time of 6 months.

The SNP rs7551288 was not associated with melanoma risk. This was assessed in the present cohort in comparison with 1988 patients unaffected of melanoma from south Germany as part of the KORA study [23]. In addition, the absence of a melanoma risk association was confirmed in the dataset from Law et al. [12] comprising 16,000 melanoma patients and 26,000 controls (*p* = 0.49). The presence of one or two rs7551288*A alleles was not associated with pigmentation related traits, such as hair colour, eye colour, tanning response or TNC (Table 1). There was no association with other host-specific factors, such as obesity, a history of a second cancer or a positive family history of melanoma.

The association of the rs7551288 genotype with melanoma-specific data was analysed. A significant association of rs7551288*A with tumour thickness (*p* = 0.034) with the initial stage at diagnoses (*p* = 0.041) and with melanoma-specific death (*p* < 0.001) was found. There was no significant association with the histological subtype and with the presence of ulceration of the primary tumour (Table 2). 

To quantify the impact on melanoma-specific survival of the rs7551288 genotype compared to other established prognostic factors such as tumour thickness, ulceration and stage at diagnoses, univariate and multivariate cox regression analyses were performed (Table 3). Univariate analyses revealed an approximately four times increased risk of melanoma death for patients with two rs7551288*A alleles (HR 3.95 [95% CI 1.99–7.83], *p* < 0.001) compared to patients with two rs7551288*G alleles. Heterozygous patients showed a trend towards an increased risk of melanoma death (HR 1.46 [95% CI 0.76–2.83], *p* = 0.26). Patients with a primary tumour thicker than 2 mm had a 1.84-fold increased risk of dying from melanoma (HR 1.84 [95% CI 1.08–3.12], *p* = 0.024) compared to patients with a primary melanoma of ≤2.0 mm. An ulcerated primary tumour increased the risk of melanoma death 2.49-fold (HR 2.49 [95% CI 1.44–4.31], *p* = 0.001) and patients with an advanced stage at diagnosis (stage III + IV compared to stage I + II) had a 3.62-fold increased risk of melanoma death (HR 3.62 [95% CI 2.16–6.08], *p* < 0.001).

Multivariable Cox regression including all four factors ‘tumour thickness’, ‘ulceration’, ‘stage at diagnoses’ and ‘rs7551288 genotype’ revealed an independent prognostic significance of the rs7551288 A/A genotype in our melanoma patients with a more than fivefold increased risk of melanoma-specific death for patients with two rs7551288*A alleles at rs7551288 (HR 5.31 [95% CI 2.30–12.25], *p* < 0.001).

**Figure 4:**
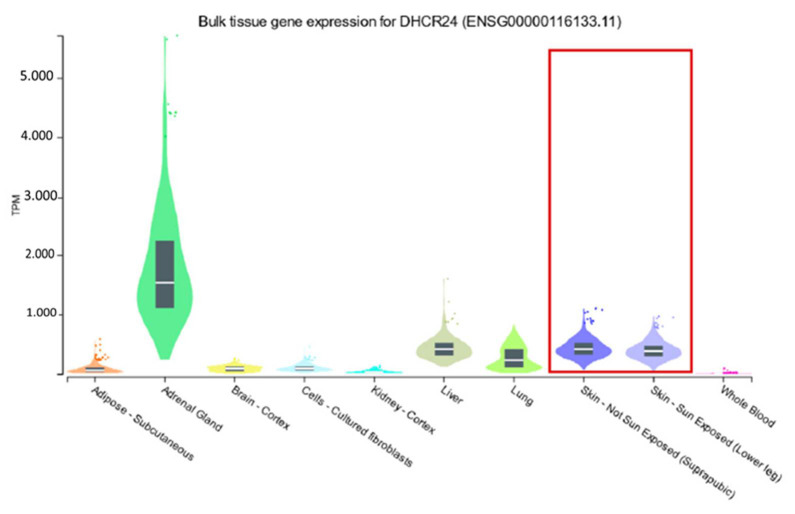
Bulk expression analyses of DHCR24 in different tissues with an accentuated expression in skin. Data from the GTex online database.

Using the public resource GTex portal [24], we reviewed the expression of DHCR24 in different tissues and found a marked expression in the skin (Figure 4). 

## 4. Discussion

The present work was conducted to investigate prognostic factors in melanoma patients aside from established tumour-specific factors. The cohort of the assessed melanoma patients was recruited within a University Hospital setting with a structured follow up routine for patients with a tumour thickness above 1 mm. Patients with tumours below a 1 mm tumour thickness had either additional risk factors or were followed up because of metastases despite their thin primaries. In a genome-wide approach with 356,384 common polymorphisms, we identified an association of the variant rs7551288 with melanoma-specific survival, independent of tumour thickness and other established prognostic factors. The SNP rs7551288*A/G is an intronic variant of the *DHCR24* gene. Carriers of one or two rs7551288*A alleles exhibited a significant impaired melanoma-specific overall survival compared to patients with rs7551288*G alleles. Searches of several data bases including The Human Protein Atlas (www.proteinatlas.org (accessed on 26 April 2022)) found no other gene immediately flanking the *DHCR24* locus expressed at a high level nor in a melanocytic-specific fashion (*MROH7*, *TTC4*, *PARS2*, *TTC22*, *LEXM* distal and *RP11-67L*, *TMEM61*, *BSND*, *PCSK9*, *USP24* proximal to *DHCR24;* www.genome.ucsc.edu (accessed on 26 April 2022).

*DHCR24* was first described in 1995 in Arabidopsis and was considered to be important for plant growth [25]. By comparison of selected brain tissues from Alzheimer patients, Greeve et al. discovered that *DHCR24* was downregulated in severely affected regions [26]. They named the protein Selective Alzheimers’s Disease Indicator 1 (Seladin-1) and found an improved resistance of Seladin-1 expressing cells through the inhibition of Caspase 3 activation, which protected the cells from apoptotic cell death. The *DHCR24* gene encodes for the 3beta-hydroxysterol delta24-reductase (DHCR24), an enzyme involved in the final step of the cholesterol synthesis [27]. A binding site for DHCR24 was found in the tumour-suppressor p53 protein [28], and the overexpression of DHCR24 was associated with impaired p53 activity [29].

The impact of DHCR24 was investigated in different types of cancer so far. High expression levels of DHCR24 in tissue specimens with urothelial carcinoma of 162 patients were found to be significantly associated with an impaired progression-free survival [30]. In cell-line experiments, the authors could also demonstrate an enhanced proliferation, adhesion and migration after enforced expression of DHCR24 and less cell viability after DHCR24 loss. DHCR24 was highly expressed in a tissue microarray with endometrial carcinoma of 258 patients, and the upregulation was associated with reduced overall survival. Silencing DHCR24 in cell lines led to a reduced metastatic ability of the endometrial cancer cells [31]. Expression levels of DHCR24 were also assessed in a study with adrenocortical adenomas and adrenocortical carcinomas and compared to normal adrenal glands. In this study, expression levels were reduced in adrenal cancer [32]. A study investigating the role of Rac1 activity in the malignant progression of sebaceous skin tumours identified *DHCR24* as a target gene. Downregulation of *DHCR24* was suggested to be an indicator for the susceptibility of malignant progression [33]. A previous study on DHCR24 and melanoma reported higher expression levels in melanoma metastases compared to primary tumours, based on analyses of cell lines, which were obtained from cutaneous metastases and the corresponding primary tumours. Upregulation of *DHCR24* was shown to be associated with resistance to apoptosis. Furthermore, the effect of the DHCR24 inhibitor U18666A was demonstrated, which increased the sensitivity of the melanoma cells against H_2_O_2_, but not against cytotoxic agents. This was suggestive for a protective role of DHCR24, specifically against oxidative stress [34]. 

To date, there is quite some pre-clinical [35] and clinical evidence available to support an effect of statins and other lipid-lowering drugs in melanoma. Statins lower human plasma cholesterol levels [36], and statin drugs might thereby abrogate the effect of high DHCR24 expression and increased cholesterol synthesis. A decreased incidence of melanoma was found in a large randomized, placebo-controlled clinical cardiology trial with lipid-lowering agents, including statins, as reviewed by Dellavalle et al. [37]. The results were later revoked by a systematic review and meta-analyses and only a subgroup analysis revealed a significant result for lovastatin use [38]. However, a subsequent prospective study of cancer incidence again found a lower melanoma risk for patients using cholesterol-lowering drugs for five or more years [39]. Another study found a lower likelihood of ulcerated melanomas in statin users, indicating a shift towards prognostic favourable tumours [40]. A trend towards a better survival in male patients with statin use was seen in a population-based cohort study [41]. A recent study by Stamatakos et al. investigated drug interactions in BRAF-inhibitor-resistant melanoma cells. The authors describe a synergistic effect of a combination treatment with the BRAF inhibitor PLX4032 and the DHCR24 inhibitor U18666A [42]. These pre-clinical and clinical findings urge further investigation of statins or the novel selective DHCR24 inhibitors [43] in melanoma patients. Our stage IV patients with the A/A genotype had the worst outcome in all treatment groups with a slight improvement under systemic treatment. Those patients might well benefit from combination treatments.

We showed an association of the intronic variant rs7551288*A of the DHRC24 gene with melanoma survival in a cohort of 556 melanoma patients. Additional studies are needed to confirm this variant as a novel prognostic biomarker. The variant has an effect on DHRC24 expression and thereby on cholesterol synthesis. We do not know if this intronic variant may also influence other genes, which could also contribute to the poor outcome of patients with the A/A genotype. However, available DHCR24 expression data in various types of cancers, including melanoma, strongly support the need for further functional studies on the *DHCR24* genotype, DHCR24 expression, cholesterol synthesis and melanoma survival. As there are some data supporting the effectiveness of lipid-lowering drugs in melanoma as discussed above, additional knowledge of the impact of *DHCR24* genotypes might help to individualize treatment.

## 5. Conclusions

The intronic variant rs7551288*A of the *DHRC24* gene is associated with impaired survival in our cohort of melanoma patients, independent of established prognostic factors. The genetic polymorphism might influence the survival probabilities of melanoma patients through an increase in Seladin-1 protein expression, which influences the cholesterol synthesis.

The variant is common and has a reported MAF of 0.37 and 0.43 in European populations. Testing for this variant might reveal a relevant subset of patients who could benefit from an intensified surveillance program and who might receive additional specific therapeutic options.

## Figures and Tables

**Table 1:** Patients characteristics and rs7551288 genotype.

Characteristics		All Patients	rs7551288 Genotype			*p* *
			A/A	A/G	G/G	
		*n* = 556 (%)	*n* = 96 (%)	*n* = 284 (%)	*n* = 176 (%)	
Gender	Male	308 (55.4)	54 (56.3)	156 (54.9)	98 (55.7)	0.971
	Female	248 (44.6)	42 (43.7)	128 (45.1)	78 (44.3)	
Age	0–30	32 (5.7)	3 (3.1)	21 (7.4)	8 (4.5)	0.502
	31–50	154 (27.7)	28 (29.2)	81 (28.5)	45 (25.6)	
	51–70	249 (44.8)	46 (47.9)	125 (44.0)	78 (44.3)	
	>70	121 (21.8)	19 (19.8)	57 (20.1)	45 (25.6)	
Hair colour	red	33 (5.9)	9 (9.4)	18 (6.3)	6 (3.4)	0.568
	blonde	239 (43.0)	38 (39.6)	125 44.0()	76 (43.2)	
	brown	242 (43.5	41 (42.7)	119 (41.9)	82 (46.6)	
	black	32 (5.8)	6 (6.3)	16 (5.6)	10 (5.7)	
	na	10 (1.8)	2 (2.1)	6 (2.1)	2 (1.1)	
Eye colour	blue	218 (39.2)	37 (38.5)	114 (40.1)	67 (38.1)	0.863
	grey	110 (19.8)	22 (22.9)	55 (19.4)	33 (18.8)	
	green	91 (16.4)	18 (18.8)	42 (14.8)	31 (17.6)	
	brown	133 (23.9)	19 (19.8)	72 (25.4)	42 (23.9)	
	na	4 (0.7)	-	1 (0.4)	3 (1.7)	
UV tanning response	no tan	97 (17.4)	20 (20.8)	52 (18.3)	25 (14.2)	0.400
	light tan	271 (48.7)	49 (51.0)	141 (49.6)	81 (46.0)	
	strong tan	130 (23.4)	20 (20.8)	59 (20.8)	51 (29.0)	
	always tan	52 (9.4)	7 (7.3)	27 (9.5)	18 (10.2)	
	na	6 (1.1)	-	5 (1.8)	1 (0.6)	
Total naevus count	0–10	134 (24.1)	18 (18.8)	77 (27.1)	39 (22.2)	0.326
	11–30	198 (35.6)	43 (44.8)	92 (32.4)	63 (35.8)	
	31–50	105 (18.9)	18 (18.8)	49 (17.3)	38 (21.6)	
	51–100	79 (14.2)	13 (13.5)	45 (15.8)	21 (11.9)	
	>100	38 (6.8)	4 (4.2)	20 (7.0)	14 (8.0)	
	na	2 (0.4)	-	1 (0.4)	1 (0.6)	
Obesity	BMI ≤ 30	426 (76.6)	77 (80.2)	220 (77.5)	129 (73.3)	0.606
	BMI > 30	100 (18.0)	14 (14.6)	53 (18.7)	33 (18.8)	
	na	30 (5.4)	5 (5.2)	11 (3.9)	14 (8.0)	
Second cancer	yes	37 (6.7)	7 (7.3)	14 (4.9)	16 (9.1)	0.212
	no	519 (93.3)	89 (92.7)	270 (95.1)	160 (90.9)	
Family history of Melanoma	yes	29 (5.2)	7 (7.3)	9 (3.2)	13 (7.4)	0.094
	no	508 (91.4)	88 (91.7)	263 (92.6)	157 (89.2)	
	na	19 (3.4)	1 (1.0)	12 (4.2)	6 (3.4)	

* Pearson Chi-Square Asymp.Sig (2-sided).

**Table 2:** Tumour characteristics and rs7551288 genotype.

Characteristics		All Patients	rs7551288 Genotype			*p* *
			A/A	A/G	G/G	
		*n* = 556 (%)	*n* = 96 (%)	*n* = 284 (%)	*n* = 176 (%)	
Tumour thickness	<1.0 mm	127 (22.8)	20 (20.8)	69 (24.3)	38 (21.6)	**0.034**
	1.01–2.0 mm	213 (38.3)	31 (32.3)	100 (35.2)	82 (46.6)	
	2.01–4 mm	126 (22.7)	22 (22.9)	73 (25.7)	31 (17.6)	
	>4 mm	53 (9.5)	15 (15.6)	26 (9.2)	12 (6.8)	
	na	37 (6.7)	8 (8.3)	16 (5.6)	13 (7.4)	
Histology	SSM	307 (55.2)	57 (59.4)	149 (52.5)	101 (57.4)	0.287
	NM	90 (16.2)	12 (12.5)	53 (18.7)	25 (14.2)	
	LMM	26 (4.7)	5 (5.2)	18 (6.3)	3 (1.7)	
	ALM	28 (5.0)	6 (6.3)	11 (3.9)	11 (6.3)	
	others	51 (9.2)	9 (9.4)	26 (9.2)	16 (9.1)	
	na	54 (9.7)	7 (7.3)	27 (9.5)	20 (11.4)	
Ulceration	no	322 (57.9)	58 (60.4)	165 (58.1)	99 (56.3)	0.914
	yes	122 (21.9)	24 (25.0)	62 (21.8)	36 (20.5)	
	na	112 (20.1)	14 (14.6)	57 (20.1)	41 (23.3)	
Stage at Diagnoses	Stage I	289 (52.0)	39 (40.6)	147 (51.8)	103 (58.5)	**0.041**
	Stage II	169 (30.4)	31 (32.3)	93 (32.7)	45 (25.6)	
	Stage III	81 (14.6)	22 (22.9)	36 (12.7)	23 (13.1)	
	Stage IV	6 (1.1)	0 (0)	3 (1.1)	3 (1.7)	
	na	11 (2.0)	4 (4.2)	5 (1.8)	2 (1.1)	
Melanoma Death	yes	64 (11.5)	23 (24.0)	28 (9.9)	13 (7.4)	**<0.001**
	no	492 (88.5)	73 (76.0)	256 (90.1)	163 (92.6)	

* Pearson Chi-Square Asymp.Sig (2-sided). Significant *p*-values shown in bold.

**Table 3:** Cox regression analyses for melanoma-specific survival.

Overall Survival					
Definition		Univariate HR (95% CI)	*p*-Value	Multivariate HR (95% CI)	*p*-Value
Tumour thickness	≤2.0 mm	1		1	
	>2.0 mm	1.84 (1.08–3.12)	**0.024**	1.5 (0.84–2.74)	0.164
Ulceration	no	1		1	
	yes	2.49 (1.44–4.31)	**0.001**	2.07 (1.15–3.73)	**0.016**
Stage at Diagnoses	Stage I + II	1		1	
	Stage III + IV	3.62 (2.16–6.08)	**<0.001**	2.37 (1.25–4.49)	**0.008**
rs7551288 Genotype	G/G	1		1	
	G/A	1.46 (0.76–2.83)	0.26	2.13 (0.93–4.86)	0.07
	A/A	3.95 (1.99–7.83)	**<0.001**	5.31 (2.30–12.25)	**<0.001**

HR hazard ratio, CI confidence interval. Significant *p*-values shown in bold.

## Data Availability

The data presented in this study are available from the corresponding author upon reasonable request.
